# A Rare Case of Levetiracetam-Induced Refractory Hypokalemia

**DOI:** 10.7759/cureus.23817

**Published:** 2022-04-04

**Authors:** Paige Coughlin, Goonja Patel, Jessica Vadaketh, Ramesh Pandit

**Affiliations:** 1 Medicine, Drexel University College of Medicine, Philadelphia, USA; 2 Medicine, Crozer Chester Medical Center, Upland, USA

**Keywords:** medication, side effect, seizures, refractory, hypokalemia, levetiracetam

## Abstract

Levetiracetam is a Food and Drug Administration (FDA)-approved drug for partial, generalized, and myoclonic seizures. Its mechanism of action as an antiepileptic involves the release of neurotransmitters from synaptic vesicles. The common side effects of levetiracetam include sleepiness, weakness, dizziness, and infection.

We present a case of levetiracetam-induced hypokalemia, which was refractory to multiple repletion attempts. A 73-year-old woman with a history of seizures, heart failure, and previous stroke was admitted to the hospital due to witnessed seizure-like activity as a result of medication non-compliance. Her serum potassium prior to the start of antiepileptic medication was 4.5 mmol/L. She was restarted on her home dose of levetiracetam 1000 mg twice daily. Twenty-four hours after starting levetiracetam, the patient was found to have hypokalemia, and the patient’s potassium levels failed to correct, dropping as low as 2.0 mmol/L despite continued repletion and normalized magnesium levels. A decision was made to switch the levetiracetam to lacosamide. Thirty-six hours after this change was made, the patient’s potassium level corrected to 3.3 mmol/L and then corrected to 3.9 mmol/L five days later without requiring further repletion. Based on her clinical course, a diagnosis of levetiracetam-induced refractory hypokalemia was made. She was discharged home on lacosamide as her new antiepileptic medication, along with a close follow-up with neurology.

Our case highlights the importance of considering Levetiracetam as a cause of refractory hypokalemia. Cases of levetiracetam-induced hypokalemia and hypomagnesemia are rarely reported in the literature, and those that have been reported vary greatly in onset and the resolution of electrolyte derangements. Given that levetiracetam is a widely used antiepileptic medication, we suggest that in cases of refractory hypokalemia, a change in antiepileptic medication should be considered.

## Introduction

Levetiracetam, brand name Keppra, is a second-generation antiepileptic medication that is Food and Drug Administration (FDA)-approved for the treatment of multiple different types of seizures such as focal, generalized, and myoclonic seizures [1]. Levetiracetam exerts its therapeutic action by binding to a synaptic vesicle protein, SVA2, which inhibits calcium release from voltage-gated Ca channels and thus decreases the release of neurotransmitters from synaptic vesicles like gamma-aminobutyric acid (GABA) and glutamine. Levetiracetam is known to have minimal side effects, which is why it is commonly prescribed by physicians [2]. Some of the most common side effects include sleepiness, weakness, dizziness, and infection [1-3]. Besides its minimal adverse effect profile, levetiracetam is also favorable due to having limited influence on the cytochrome P450 system, thus minimizing drug interactions compared to multiple other antiepileptics [2].

Hypokalemia is one of the most common electrolyte disturbances that physicians come across, and it can have multiple adverse effects on different systems of the body [4]. The most notable and life-threatening effect is that it can cause life-threatening arrhythmias and affect cardiac muscles. The neuromuscular system is also affected in hypokalemia and can cause severe muscle weakness, which can affect respiratory muscles, leading to difficulty in breathing, as well as muscles in the gastrointestinal tract, leading to nausea and vomiting [4]. Lastly, hypokalemia can affect kidney function and reduce their ability to concentrate and filter substances. Thus, hypokalemia should be recognized and treated timely to avoid multiorgan dysfunction.

Herein, we present a case of levetiracetam-induced hypokalemia, which was refractory to multiple repletion attempts.

## Case presentation

A 73-year-old woman with a history of seizures, heart failure, and previous stroke was admitted to the hospital due to witnessed seizure-like activity as a result of medication non-compliance. The patient had been on levetiracetam for several years prior to her current admission. As a result of her severe postictal state, she was intubated for airway protection and transferred to the intensive care unit. The patient was extubated after 24 hours with no major clinical events. Her serum potassium on the day of presentation, prior to the start of the antiepileptic medication, was 4.5 mmol/L. The potassium levels over the hospital course are detailed in Table 1. The patient was restarted on her home dose of levetiracetam 1000 mg twice daily. Twenty-four hours after starting levetiracetam, the patient was noted to have a serum potassium level of 2.7 mmol/L and mild hypomagnesemia with a serum level of 1.5 mg/dl on morning lab work. At this time, her magnesium and potassium were adequately repleted utilizing the intravenous as well as oral routes, and the patient’s home furosemide dose was held to avoid further hypokalemia. The patient's magnesium levels were within normal range after replacement with 2 gm intravenous magnesium sulfate, but the potassium levels failed to correct, dropping as low as 2.0 mmol/L despite daily intravenous and oral potassium repletion based on the lab results. Magnesium levels were within normal limits of 1.8 to 2.4 mg/dl from Day 2 onward.

**Table 1:** Measured potassium results over the course of hospitalization

Hospitalization Day	0	1	2	3	4	5	6	7	8	Reference range
Potassium Level (mmol/L)	4.5	2.79 & 2.0	2.9 & 3.6	3.0 & 3.4	2.7 & 3.3	3.3	3.3	3.3	3.9	3.6-5.2 mmol/L (SI units)

The patient was noted to have normal renal functions, and urine analysis was negative for any significant urine potassium loss. There was no diarrhea or vomiting noted suggestive of any excess gastrointestinal losses, and the patient was tolerating the cardiac diet with fluid restriction. On hospital day four, a decision was made to switch the patient’s evening dose of levetiracetam to lacosamide. Thirty-six hours after this change was made, the potassium level rose to 3.3 mmol/L. Her potassium remained at this level for four more days without further repletion, with the intention to monitor for any further recurrence of the severe hypokalemia. Potassium levels then corrected from 3.3 mmol/L to 3.9 mmol/L five days after the switch of medication and one-time potassium repletion with 40 mEq of potassium orally. Based on her clinical course, a diagnosis of levetiracetam-induced refractory hypokalemia was made. She was discharged home on lacosamide as her new antiepileptic medication, along with a plan to closely follow up with Neurology as an outpatient.

## Discussion

Levetiracetam is one of the most commonly prescribed anti-epileptic medications as primary or adjunct treatment [1,5]. Levetiracetam use is associated with lethargy, nausea, and drowsiness, but it is not commonly known to be associated with hypokalemia. A PubMed search for “Levetiracetam and Hypokalemia” returned two articles featuring adult patients with this presentation, as summarized in Table 2.

**Table 2:** Literature Review Findings Case

Research Article	Sex	Age	Levetiracetam Dose	Potassium (mmol/L)
Aksoy D et al., 2014, [6]	Male	23	1g/day PO	3.1
Vallianou N et al., 2015, [7]	Female	79	1g/day IV	2.43
	Female	90	1g/day PO	2.44

Like our patient described above, these patients too were on levetiracetam for seizure prevention and developed hypokalemia, which subsequently resolved after this medication was discontinued [6-7]. Cases of levetiracetam-induced hypokalemia and/or hypomagnesemia are rarely reported in the literature, and those that have been reported vary greatly in onset and the resolution of electrolyte derangements. For example, the cases above differed in symptom onset from our patients'. Durdane A reported a case of hypokalemia within one month after beginning levetiracetam for epilepsy treatment [6]. Vallianou et al. reported two patients who were elderly females, like ours, who developed hypokalemia within a few days of initiation of levetiracetam [7].

In our patient, other common causes of hypokalemia were considered, however, none were as directly implicated as levetiracetam. The patient did not have any history of recent vomiting or diarrhea, diabetes, insulin use, or renal disease that could lead to inappropriate potassium loss. A review of medications that our patient was taking at the time of hypokalemia development showed: Cardizem, Pepcid, Pradaxa, Unasyn, and levetiracetam. Furosemide could lead to further potassium loss and contribute to hypokalemia. Our patient was taking furosemide, and the patient’s hypokalemia persisted in spite of holding furosemide. Additionally, all other medications were maintained following the change from levetiracetam to lacosamide for seizure control, after which the hypokalemia resolved.

## Conclusions

Our case highlights the importance of considering levetiracetam as a cause of hypokalemia refractory to regular supplementation in a select patient population, and in cases of refractory hypokalemia, a change in antiepileptic medication should be considered. Though the mechanism of action of this condition is unclear, the literature and our experience present a concern for hypokalemia with levetiracetam use. Further studies are needed to determine the relative risk of this condition and outcomes for patients.
